# Addressing the Language, Education, and Identity Development of Transnational Youth in Preservice/Inservice Teacher Education Programs Using Forum Theater

**DOI:** 10.3390/bs16020256

**Published:** 2026-02-10

**Authors:** Theresa Catalano, Tianna L. Bankhead, Amanda R. Morales, Crystal Bock Thiessen, Brendan A. Kachnowski

**Affiliations:** Department of Teaching, Learning and Teacher Education, University of Nebraska-Lincoln, Lincoln, NE 68588, USA

**Keywords:** Forum Theater, arts-based education, transnational youth, identity, language, preservice/inservice teachers

## Abstract

Substantial research has been dedicated to the study of transnational students’ language, education, and identity development in order to cultivate their funds of knowledge and to improve their schooling experiences. However, as this Special Issue points out, a more holistic and transdisciplinary approach is needed. The present paper does just this, zooming in on teacher education programs designed to prepare teachers to work with transnational students using transdisciplinary arts-based approaches, and in particular, Augusto Boal’s Forum Theater. Employing collective autoethnography and building on a larger research study that explores participant reflections on experiences engaging in Forum Theater, we reconsider three scenarios from arts-based workshops conducted with transnational learners and preservice/inservice teachers (aka teacher learners). In doing so, we deconstruct exactly how the exploration and brainstorming of effective responses to transnational youth experiences of discrimination, stereotyping, and racism in the Forum Theater workshops are harnessed to help participants understand the psychological, cognitive, and behavioral factors at play that impact transnational youth language, education, and identity development. Findings show how each scenario could lead to greater understanding of transnational youth experiences, and the development of teacher learners’ critical consciousness in working with these students.

## 1. Introduction

Transnational students can be defined as youth “who live across two or more nations and who attend school in one or more of the nations they call home” (Skerrett, 2020, p. 499). This term is associated with students who settle either permanently or for long periods of time in a new country (J. Kim & Richardson, 2018), and is often connected to the work of Zúñiga and Hamann (2009) (and others) who studied learners moving back and forth between Mexico and the United States for a variety of reasons. Since then, use of the term has expanded to include a variety of lived experiences such as that of students who may have been born in their parents’ destination country but never lived or visited their country of origin (but still identify culturally and/or linguistically with this culture). Others referred to as ‘transnational students’ study in other countries before migrating but keep close ties with their countries of origin and tend to “acquire practices that distinguish them” across the various cultures they have been exposed to (J. Kim & Richardson, 2018, p. 3). Instability and mobility tend to be dominant forces in these students’ lives and many of them (but not all) end up being identified as ‘English learners’ (or multilingual learners) in U.S. schools or given similar names in other countries which signal that they need support in the language of schooling if such programs exist. Rather than taking a deficit perspective that characterizes these students as lacking or ‘limited’, we align with scholars such as Kwon (2022, p. 20) who believe we must reframe transnational youth as “transnational and multilingual experts” whose linguistic, cultural, and experiential knowledge gained through the crossing of borders and boundaries should be framed as valuable assets and resources.

Despite the growing number of transnational students in educational systems worldwide (Zúñiga & Hamann, 2009) and years of research and attention to their schooling experiences, these students often still face discrimination, negative stereotypes, racism, and inequitable learning opportunities. Responding to this Special Issue’s call for holistic, transdisciplinary research that centers the funds of knowledge and resource development of transnational youth, we focus our analysis on teacher education programs and how they can better prepare teachers to work with this distinct population of students via one particular arts and community-based approach: Forum Theater.

Building on a previous study (Morales et al., 2025), this paper explores three scenarios created by preservice/inservice teachers (aka teacher learners) in Forum Theater workshops implemented in a teacher education program at the University of Nebraska-Lincoln in 2022 and 2025 in order to understand how the exploration and brainstorming of solutions in transdisciplinary Forum Theater workshops can provide a more holistic way to inform teacher learners of the psychological, cognitive, and behavioral factors that shape transnational youth’s language, education, and identity development. We draw on Anzaldúa (2002) and Przymus and Serna Gutiérrez (2022) to bridge an understanding of the in-between, liminal spaces that transnational youth often frequent into “more equitable teaching and life experiences” and as a way of “mythifying” transnational youth as “synonymous with survival, intelligence, multilingualism, and interculturalism” (Przymus & Serna Gutiérrez, 2022, p. 147). Using the teacher learner created scenarios, we explore how the embodied enactment of and reflection on each scenario fostered critical consciousness raising among participants and better prepared the preservice/inservice teachers (aka teacher learners) to work with transnational youth. We begin with a brief review of studies on transnational youth and teacher education in this area.

## 2. Literature Review

Educators that work with transnational youth in the public school system are tasked with creating environments that are effective and responsive to their learning through incorporation of their experiences, culture, and linguistic backgrounds (Ganesan & Morales, 2023). The classroom is a space for students to build trusting relationships with their teacher and their peers. When authentic connections are built and trusting relationships are fostered, the student’s affective filter is lowered, and space is created for them to take academic risks (Weinstein & Novodvorsky, 2014). However, it is quite common for transnational and multilingual students’ sense of belonging to be undermined by the frequent “othering” they and their families experience (Borrero et al., 2012), which can affect students’ psychological, behavioral, and cognitive processes (Walton & Crum, 2021). Furthermore, students’ abilities to navigate, resist, and overcome such negative experiences influence their identity development in significant ways.

Focusing on the United States, which is the context for our study, transnational youth often have the experience of being caught between two worlds—tasked with navigating the obligatory travel to their home country or cultural duties and navigating the “Americanization process” (Sánchez, 2001). First- or second-generation immigrant youth experience racialization (Kiramba et al., 2025/2022) that is very specific to the context of the United States while subconsciously navigating pressures and making decisions on whether they should be assimilating or acculturating (Birman & Addae, 2015; Kiramba et al., 2025/2022) to the dominant society. Because of this, they may engage in risky behavior (Bui, 2015) and/or struggle to identify their race despite being aware of their ethnic heritage (Schwartz et al., 2015). In turn, they grapple with two questions. (1) How to behave based on the ways they are perceived by others and (2) Why they must respond to these external expectations and assumptions of who they are.

Take for instance the study by Schwartz et al. (2015) and the example of Yuliana. In her developmental years, she was proud of her Dominican and New Yorker identity. She developed friendships with other Caribbean students that supported her stance on who she was and where she is from. However, when she began college and interacted with people that wanted to know where she was “really from” she began to question how she should identify racially. Asking herself: Am I Latina? Black? Dominican? A New Yorker? (Schwartz et al., 2015). This psychological process of questioning who you are and how you fit within particular social, cultural, and geographic contexts ties directly into identity development. When transnational youth have a strong sense of ethnic identity it “promotes well-being and protects against anxiety, depression, behavior problems, and substance use” (Schwartz et al., 2015, p. 155). Therefore, these youth need intentional, supportive spaces to make sense of their experiences as they navigate multiple, unfamiliar, and often marginalizing contexts within the U.S.

In a behavioral sense, we can look at the work of Bui (2015), who documents how immigrant youth who are navigating such complexities often are surviving rather than thriving. She indicates that when youth “are more acculturated to the dominant culture they may be more vulnerable to the effects of discrimination because they are less aligned with their traditional culture and more sensitive to negative attitudes about and stereotypes of their group” (p. 245). When an outlet or a space has not been created for immigrant youth to discuss and pushback against discriminatory policies or behaviors from peers and educators, there is a higher chance that they will internalize these messages and turn to crime or substance abuse as a form of protection, coping, and self-preservation (Bui, 2015). Another study conducted with African immigrant youth found that students often face the challenge of isolation in their schools due to discrimination and the marginalization of their race, language, and/or culture (Kiramba et al., 2025/2022). Part of this isolation can be related to moving through the world in racialized bodies perceived as threatening. One student, for example, shared that peers’ perceptions of his body and masculinity as a threat hindered his ability to make friends.

Language development and hierarchies are another aspect of isolation that these students experienced. These youth felt that their language development made it difficult to make friends outside of the English language learners’ classroom and therefore they sought to ascribe to assimilation in terms of language use (Kiramba et al., 2025/2022). Furthermore, they recognized that there are clear language hierarchies and observed that their home languages were unknown by their peers and their teachers, and therefore, of less value. While these students valued their native languages, they knew that multilingualism was not necessarily valued in the dominant culture (Kiramba et al., 2025/2022), causing them to question their identities.

The experiences of transnational youth documented here are common occurrences in our public school system. Yet these highly capable youth are participants in our schools with a variety of cultural, linguistic, and experiential knowledge that can and should be utilized in their education. However, when there is not a space for transnational youth to work through the discriminatory practices that their peers and educators often engage in, they are at risk of internalizing this othering.

In the context of teacher practices for working with transnational youth, several studies identified Forum Theater as a tool for fostering critical consciousness (*conscientização*) and addressing the discrimination/Othering that transnational youth often experience. Critical consciousness is about learning how to identify injustices within society and how to take action against them (Freire & Macedo, 2005). Caldas (2018), for example, used Forum Theater as a liminal space in which bilingual teachers could discuss conflict and challenges in the highly politicized world of bilingual education. She used real scenarios based on situations that happened to bilingual teachers for the teacher learners to act out, arriving at a *moment of crisis* when they felt unsure of how to respond. Then together as a class, the *spectactors* (as audience participants are referred to in Boal’s work) gave suggestions and helped the teacher learners build confidence to respond in more productive and socially just ways. This same idea was manifested in the context of preparing undergraduate preservice teachers and practicing teachers who were graduate students to work with transnational youth in a study by Morales et al. (2025). In this study, the authors engaged students in activities centered on “trust and community building” and asked students in mixed groups (which included transnational youth and those identifying as international students or migrants/refugees) to brainstorm microaggressions (e.g., incidents of racism, linguicism, discrimination, stereotyping) they had witnessed or experienced to then act out, discuss, and propose more just and agentic responses. The present paper builds on this study, focusing attention on the actual scenarios that teachers created and how these contributed to their learning. The next section describes our theoretical framework on teacher education for transnational youth which is grounded in arts and community-based approaches to learning.

## 3. Transdisciplinary Arts and Community-Based Approaches to Teacher Education and Research

Arts and community-based (ACB) research such as ours is rooted in approaches to teaching and learning that integrate aesthetic experiences in education across multiple levels (Bowen & Kisida, 2017). Aesthetic education is about fostering appreciation, reflection, cultural understanding, and participatory engagement with the arts (D’Olimpio, 2022; Greene, 2001). The opposite of ‘anaesthetic’ (Greene, 2011), this type of pedagogy is designed to awaken people from set ways of thinking (such as deficit views of transnational youth) and to provide more embodied understandings of complex issues (Macintyre Latta, 2013). In this way, ACB approaches provide a more holistic and transformative model for teacher education that is often transdisciplinary (Morales et al., 2025). By transdisciplinary, we mean working across disciplines and content areas (e.g., behavioral sciences, multilingualism, aesthetic education, TESOL [Teaching English to Speakers of Other Languages], immigration studies) to explore real-world issues that can be understood differently and that can spur more practical solutions. By integrating multiple disciplines and including participants from a range of backgrounds and programs (particularly those connected to marginalized spaces where transnational youth often reside) we gain a more comprehensive, multi-perspective understanding of our topic. Findings from transdisciplinary ACB research show us that aesthetic experiences in teacher education can have lasting impressions on teacher learners and facilitate the disruption of long-held beliefs about themselves and the multiple and intersecting identities of their students (Powers & Duffy, 2016).

Our study is also influenced by arts practice research (APR) (Phelan & Nunan, 2018) that advocates for the arts having a deep (and varied) role in the research process (see Section 4.1). As mentioned above, we focus on one approach to ACB and arts practice research: Forum Theater. Forum Theater is just one part of Boal’s (1979) *Theater of the Oppressed* which, highly influenced by Freire’s (1970) *Pedagogy of the Oppressed*, demonstrates how theater can be more than just drama-based art, it can be a space of resistance. As a sensorial way of transmitting knowledge, theater can incite action, becoming in a way a “rehearsal of revolution” (Boal, 1979, p. 155). We now turn to how exactly we transformed theater into action in our workshops, providing details on our methodology and the workshops themselves.

## 4. Materials and Methods

### 4.1. The Methods

As part and parcel to the transdisciplinary nature of this study, we act as “methodological scavengers”, “seeking out methods appropriate to the analytical task regardless of their disciplinary home” (Burnett, 2025, p. 31). First, as described in the previous section, we engage in arts practice research (APR) which positions all our experiences with Forum Theater as data. In this way, scenarios that participants acted out as part of the Forum Theater workshops become sites of study, of reflection, and of analysis related to our research question. In APR, the artistic component can play a key role in the research methods, constitute the entire work, or just serve as the data. By using APR as part of our framework, we recognize and intentionally position art in and of itself as a “site of meaning-making and knowledge construction” (Phelan & Nunan, 2018, p. 3).

Secondly, we utilize both sequential and concurrent collaboration models within collective autoethnography (Chang, 2013) to “systematically analyse” our experiences as they are embedded in the larger social and cultural context of teacher education (J. H. Kim, 2015, p. 123). Collective autoethnography allowed us as a group to talk about our own experiences as data (self-observational, self-reflective, and self-analytical) as well as our interactive engagement participating in and leading the Forum Theater activities as data, focusing on three scenarios that stood out in our minds as particularly helpful for critical consciousness raising among teacher learners.

### 4.2. The Players

All the participant researchers (as referred to in autoethnography) were co-leaders and/or participants in the Forum Theater workshops at some point over the last six years, and all are faculty or doctoral students at the University of Nebraska-Lincoln. Our institution is a land grant public university which serves the state of Nebraska. Nebraska, like many Midwestern states, has experienced marked demographic changes in the last thirty-five years and despite having a population that includes 18.2% of K-12 students identified as ‘English learners’ (Nebraska Department of Education, 2025) of which most could be considered transnational youth, schools served by our university often struggle to meet the needs of these students. Moreover, there is a lack of teachers that match the demographics of the students, or that understand transnational experiences.

Participant researchers (and co-authors) were Tianna (doctoral student and instructor of multicultural education), Brendan (doctoral student and instructor of multicultural education), Crystal (instructor of transnational learners [aka international students] in the Intensive English Program), Theresa (professor of language education/linguistics), and Amanda (professor of multicultural education). For all scenarios, we use excerpts from discussions (aka reflection sessions) with these participant researchers recorded on 10 October 2025 (more details can be found in our Section 4.4). All participant researchers have lived and worked in multiple countries but do not consider themselves transnational learners. However, they are all experienced teachers of transnational youth and recognize the great need for teachers to better understand how to meet the needs of these students.

In our second and third scenarios we draw on a 22 February (2022) iteration of the workshops, and we include excerpts from teacher learners in these workshops in the third scenario, while concentrating on our own processing and analysis of these workshops in the second scenario. Participants in these workshops were either preservice teachers from a multicultural education course, graduate students (and practicing teachers) from an intercultural communication course, or transnational learners from the university’s intensive English program.

In the case of preservice teachers in the multicultural education course, there were 21 predominantly white (domestic) undergraduate elementary or secondary education students in various subject areas whose future students would surely include transnational youth. There were also two transnational (aka multilingual international) students: one from Korea (who spoke Korean and English) and one from Guatemala (who spoke Spanish, K’iche’, and English). This group was taught by Amanda.

The participants in the intercultural communication course were all graduate students, of which the majority were practicing teachers. Most were language teachers in K-12 settings or higher education who had at least a few transnational youths in their classes, and most were pursuing their master’s or doctorate degrees. There were 11 teacher learners in this class, many of whom identified as transnational and originated from various countries such as Morocco, Burkina Faso, Argentina and Spain, China, and the United States. There were also American students in the class including five white students who specialized in working with transnational students (but wanted to continue to grow as teachers), one Latina, and one Ho-Chunk (Native American) woman. Languages spoken in this group included Ho-Chunk, Mooré, French, Arabic, Spanish, Mandarin, Cantonese, and a variety of World Englishes. Theresa was the instructor of this course.

The last group of participants in this intentionally heterogenous workshop was made up of seven transnational learners in our university’s Intensive English Program (IEP) which serves pre-matriculated students. These students were originally from Sudan, Honduras, and Brazil. All seven were enrolled in an “English for Campus and Community Engagement” course as part of their IEP which was designed to provide them with ample practice of English with local peers. The Forum Theater activities were integrated into the IEP course and Crystal was their instructor. Like teacher learners in the other groups, this was their first experience with Forum Theater. Although we have described this group here because they were part of the workshops, excerpts from reflections have not been included in this paper since they provide the central data for another study that is currently in press.

Scenario one describes a different iteration of the workshop which occurred on 7 June 2025, in a doctoral seminar on arts and aesthetic education for multilingual learners. In this workshop, there were eight doctoral students who were all practicing teachers that were interested in working with transnational youth. Theresa led this workshop and Brendan and Tianna were also students in the class. They drew on this iteration of the workshops because they were active participants in creating and performing this scenario, and it was fresh in their minds and bodies. The data from this scenario come only from our discussion on 10 October 2025, which includes insights from the three participant researchers as they reflected on performing this scenario together, and hence we will not describe the other participants in the workshop here.

### 4.3. The Workshops

The Forum Theater workshops have been offered once or twice a year since 2019 (with a brief stop in 2020 during the pandemic). As a vital component of the model, the workshops are often hosted in collaboration with the university’s Intensive English Program (taught by Crystal). In an effort to foster reciprocity, the workshops are designed to provide important opportunities for international students (as community partners) to interact with students who were highly proficient in English and to build relationships with domestic students at the university.

Before the workshops began, participants read or viewed materials on microaggressions. We prioritized this topic because microaggressions frequently affect transnational youth and students of Color, especially in classroom settings. By engaging with resources on this topic, teacher-learners could better understand the harm microaggressions cause and engage in deeper conversations around strategies to address them more effectively. Each three-hour workshop was held on the University of Nebraska-Lincoln campus within walking distance for students. We began by warmly welcoming the participants, framing the workshop, and inviting them to reflect on shared agreements for creating a brave and inclusive space (Singleton, 2015). This was followed by numerous getting-to-know-you games and warm-up activities adapted from Boal (1992/2002), necessary to build the kind of environment in which participants could lower their affective filters and feel comfortable enough to share their experiences within their groups.

For the Forum Theater activities, instructors modeled a scenario for participants so they could see how the activity would work. Then participants were instructed to brainstorm and discuss microaggressions they had heard about, witnessed, or experienced, choosing one as a group to re-enact together. Each group was then given time to practice in their own space, gathering props or other things needed to perform the scenario. Once all groups were ready, they performed their scenes, stopping at the point where the tension/conflict or *moment of crisis* occurred (Boal, 1992/2002). The audience of ‘spectactors’ were then asked to brainstorm possible responses and engage in dialog together about which responses might be the most effective or which they would personally feel comfortable with. These suggestions were re-enacted by each group, allowing the audience to witness a more agentic and effective response to each scenario. The workshops ended with critical reflections and a whole group dialog on the overall experience and what participants gained from it. As ‘homework’, participants were then instructed to write about their experiences and what they learned about working with, and advocating for, transnational youth in the process. Although all participants completed this reflection assignment, for the purposes of this paper we include excerpts (in scenario three) only from the teacher learners (and not the transnational learners from the IEP) since we have a separate paper in progress that describes their reflections.

### 4.4. The Data

As described above, multiple types of data were collected for this study. Written reflections from the participants (teacher learners’) about their experiences in the workshop were collected after the 22 February workshop on 1 March 2022. We gained Institutional Review Board (IRB) approval to gather these data. Participants were informed of the study’s purpose, their right to withdraw at any time, and the data collection methods, including the use of deidentified excerpts from their reflections in publications and presentations. In order to ensure confidentiality and anonymity and to minimize risk because topics in the scenarios were of a sensitive nature, protecting the identities of teacher learners whose quotes are included in this paper was critical. As such, although we included details about these participants, we anonymized our reporting of the excerpts so that comments cannot be attributed to specific participants. All digital data (e.g., recordings, transcripts) were stored on password-protected, encrypted drives.

As described, principal data used for this paper were drawn from a two-hour collaborative reflection session with participant researchers (who are also co-authors) at a local coffee shop on 10 October 2025. Before the session, we each gathered memories, notes, and observations from workshops we have participated in, choosing one memorable scenario to share that might lend itself to answering our research question. In this collaborative ‘reflection session’ we discussed what we saw and heard and how this helped answer our research question. All sections of our findings include reflections from this discussion.

Our collective discussions helped us offer an insider perspective on the value of Forum Theater and arts-based activities as a whole and provided a safe intellectual space for us to bounce ideas off each other, and spur dialog that connected our experiences to research in this area. Tianna recorded and transcribed the conversation on Zoom and from this, we extracted quotes from the participant researchers about what was gained in the workshops, as well as details about each of the three scenarios acted out by participants in the workshops. All quotations in our analysis come from this discussion except for some quotes from written reflections of teacher learners in scenario three. These quotations from workshop participants have been labeled ‘written reflection(s)’. Table 1 below provides a clear mapping of each scenario.

### 4.5. The Analysis

Part of the data analysis occurred in the reflection session when participant researchers discussed the scenarios and brainstormed how they might contribute to our research goals. Theresa then took these initial conversations from the transcript and wrote an initial draft using a “layered account” which juxtaposed our experiences with quotations from students and participant researchers (talking about the data) contextualized by our theoretical framework and literature review (J. H. Kim, 2015, p. 209).

An initial draft of the paper was then created, and all participant researchers added their thoughts, additions, and comments to the drafts before each revision. What follows is a description of the three scenarios that were chosen as representative of the types of situations that participants worked through and the overall thematic breadth of the workshop experience, all of which was based on real events as described and reenacted by participants. After each description we include implications for the psychological, cognitive, and behavioral decisions that impact transnational youth language, education, and identity development from our analysis as well as an explanation of how teacher learners were able to learn these things from the scenarios.

## 5. Findings

### 5.1. Scenario 1: Reimagining Teachers of Transnational Youth: The Classroom

This scenario is from a version of Forum Theater Tianna, Brendan and Theresa participated in on 7 June 2025 and which Tianna and Brendan recounted in the reflection session. All examples in this section come from this reflection session. In this graduate class, Forum Theater was used to help practicing teachers better understand situations that transnational youth encounter. In the scene, participants recreated an elementary classroom in which Brendan was identified as an “English learner” (drawing on Brendan’s own past experiences) and he played his younger self at a more advanced level of learning English than his peer. His peer acted the role of his friend who had just arrived from Mexico, and Theresa and Tianna were spectactors. The scene began with Brendan’s friend trying to understand the teacher who was speaking very quickly and not acknowledging him or recognizing that he might need help or some multimodal cues or scaffolds in order to understand. Not only did the teacher ignore these issues completely, but she also became angry when Brendan tried to help his friend by translating for him. Speaking in a loud voice in English, she told Brendan, “Don’t translate for him, let him learn. Don’t help him”. This moment then became the *moment of crisis* in which the actors stopped and asked the spectactors to reimagine the teacher’s role so that it was less harmful to both students involved. That is, how could we change the teacher’s behavior so that Brendan and his friend could have had a better learning experience? After much discussion and brainstorming together as a group, it was decided that Theresa would reconfigure the role of teacher by beginning class in Spanish (the home language of the learners) and letting Brendan and his friend know what they were going to do while encouraging Brendan to help his peer with any words he did not know. She also let them know where bilingual resources were to help them access the content before moving to instruction in English. These actions of the teacher broadened access and intentionally flattened the language hierarchy, changing the behaviors of both Brendan and his friend who appeared happy and relaxed as compared to the previous scene in which they had expressed anger or fear. We now turn to the implications of this scenario.

### 5.2. Implications

Our discussion touched on the trauma of the situation for both the student and the teacher, especially because the teacher was not properly prepared for this situation. Brendan reflected on his feelings as he played the role of the student, realizing afterwards that the role play was not far from his own experiences as an English learner. He says: “I mean now, reflecting, as the student… [I was] not only reprimanded or disciplined for helping a student but also, like, my language was critiqued in that moment”. This comment reminded us of this quote from Anzaldúa (1999):

“*So, if you want to really hurt me, talk badly about my language… I am my language. Until I can take pride in my language, I cannot take pride in myself*”.

(p. 81)

Hence, not being able to use his language to help his peer had the psychological effect of trauma, damaging his identity and sense of self. This is because at the crucial time when Brendan was trying to figure out who he was and how he fit in (Schwartz et al., 2015), the fact that he was not allowed to use his language (especially as part of who he was as a caring person), spoke volumes about the teacher’s devaluing of his language and culture, and sent him into an identity crisis.

Brendan later realized that enacting the scene in the teacher education context allowed him “space to understand what had happened and when it happened” in his life (context), which is something we pointed out in our literature review as an essential element that teachers must understand about their transnational youth. This newly developed critical consciousness of injustices from his own past, and concrete visualizations of what social justice for him might have looked like gave him hope that restorative justice can be possible “when non-marginalized people are constantly critically thinking”. We also talked about the impact of this kind of situation on student behavior. Tianna commented in the reflection that:

*I think we discount students’ percept… not perceptions… the way they can feel the vibe, you know what I mean?… They’re able to read the room, and they know when their teacher is not… welcoming, or not supportive, and so… I think some students, they’re gonna withdraw, but I also think that’s also where we get defiance, and students that don’t want to do the work, or they don’t want to practice their English. They’re like, why would I?… They can, especially if it’s… you know, a situation where they switch classes and they know, like, oh, I cannot stand going to second period, so I’m gonna do what I can to get kicked out of class, so I don’t have to be in there.*

We then pondered how the actions of the teacher created a hostile environment, as noted by Brendan:

*While we… think of withdrawing from the classroom as a bad thing, I also think it’s their form of justice in that moment, right? It’s like, I’m gonna withdraw so I have access to my language, I have access to my peers. You’re not giving up their identity. Yeah, like… hold on to it through withdrawing…*

Our conversation then turned to the way in which teachers reproduce ideologies they hear in the media and around them. During the group brainstorming of alternative actions the teacher could have taken as a group, the audience spectactors considered the underlying assumptions of the teacher. Why did they act the way that they did? In what ways was the teacher reproducing ideologies that circulated in the wider society in the classroom? How could these actions of the teacher have a ripple effect in the classroom in terms of how other students treated transnational youth? That is, would they mimic the disdain and ignorance of the students’ needs? Would bullying of these students occur? Would they “make fun of the child… exclude the child” (Tianna), etc.

We also discussed how reimagining the role of the educator together as practicing teachers helped them disrupt what they thought was possible. When Theresa stepped in and began class in Spanish this made the teacher learners consider the possibility of learning the language of their students, or at the very least, reflect on and discuss how their own actions and expectations can unintentionally cause conflict or harm. It also made them consider their actions from the perspective of the learner. In doing so, arts-based (and social justice oriented) activities such as Forum Theater might result in “better learning” on the part of students of the teacher learners and provide a model for restorative justice conversations that teachers might have with their own students.

### 5.3. Scenario 2: Raciolinguistics and Belonging: The Bus

In this scene, which was performed by teacher learners in the February 2022 iteration of Forum Theater and was re-told by Crystal, three participants enacted riding the bus, and one was the driver. In the performance, two of the students were transnational students (which was true to their own identity), and one played the role of a US born and raised Muslim woman wearing a hijab. In the scene, they were sitting together and chatting in English during the ride when at a certain point, the bus driver turned to the Muslim woman and asked her something in Arabic. She responded that she did not speak Arabic, but he continued to talk, and then finally when he realized she did not understand him, he made a comment about her lack of proficiency in Arabic. This was the *moment of crisis*, and the point at which the scene stopped, and the spectactors were asked to discuss the microaggression and what was problematic about it. After the discussion, spectactors reconfigured the scene so that the bystanders (the other international students on the bus) stood up for the woman, telling the driver that just because she was Muslim, and wore a hijab does not mean that she speaks Arabic. We now turn to our discussion of the implications of this scenario for teacher learners noting that examples in this section are also exclusively from our collective reflection session.

### 5.4. Implications

In our discussion of this scenario, Crystal immediately mentioned that some of her transnational students were shocked because they did not know that assuming you spoke a language was a microaggression. “They’re like, why would that be a… why would that be something offensive? I’d like to think that somebody speaks Arabic” (Crystal). At that point in the workshop, we took the time to break down what happened referring to raciolinguistics which provides the theoretical basis for understanding how speakers’ racialized groups can be important factors in “evaluating the speaker’s language appropriateness” (Chávez-Moreno, 2024, p. 255). That is, because she looked (and dressed) like an Arabic speaker, the driver assumed she was. Crystal pointed out that at the heart of this scenario was this issue of identity because even though the subject of this scenario was born and raised in the United States, she was somehow considered “not really American”. Below, Tianna underscores the psychological effects for transnational youth when they experience these microaggressions in public spaces, saying that they come to school feeling:

*Like, they don’t belong, with maybe, like, their peers that share the same religion and potentially the same ethnic, racial identity, but they don’t have the linguistic connection to them.*

This desire for belonging (and lack of) has been documented as one reason that transnational youth often feel depressed and consequently disengage at school (Catalano, 2016). Another issue that came up in the discussion related to the way that transnational youth sometimes experience this in-betweenness—neither belonging in their parents’ country of origin nor their own—finding it difficult to “take pride in their own language practices and identities” (Przymus & Serna Gutiérrez, 2022, p. 133). Often not having the opportunity to learn or further develop their home language because of monolingual/white supremist ideas about education and the hegemony of English, they are under-confident in their home language abilities or lose the language completely. Sometimes referred to in bilingual Spanish English communities as “No Sabo Kids” because they are of Latino descent but cannot speak Spanish fluently, they often become the butt of jokes or “language shaming” by relatives or peers (Ayala-Saracay, 2025). Ayala-Saracay (2025) found that adverse experiences such as being assumed to speak a language (and consequently derided because of it, such as in our scenario) can influence identity, feelings of belonging, and even language proficiency.

This discussion of the psychological harms of raciolinguistic microaggressions led to a dialog on how conversations in the Forum Theater events can sometimes lead to people who have been the perpetrators of similar microaggressions in the past changing their own behavior in the future. In this way “for non-marginalized people in these scenes, it allows them to unpack towards restorative justice, but for the marginalized, it allows us to unpack towards healing” (Brendan). We also deliberated the nuances of this kind of teacher education and the way in which the current political climate requires us to handle these situations with care. That means helping teachers understand symbolic or metaphorical violence, such as in the microaggressions, as opposed to physical violence. Crystal argued that we must be careful because “a lot of people don’t think they did anything wrong because they were nice”. Then when we use terms like “symbolic violence” to talk about the microaggressions:

*… that actually triggers reactions of, you know, ‘I’m a nice person, why are they calling me racist? Why are they calling me violent?’*.

(Crystal)

Brendan noted that the use of impactful pedagogies such as Forum Theater in teacher preparation are vital in that they provide space and time to help teachers understand how psychological violence and behavioral violence is occurring at each level, as well as:

*… how do we reroute conversation and action towards restorative practices, restorative justice, where people are unpacking the trauma that they are creating or inheriting at each level?*

ACB allows for this unpacking to happen when we may not have the words to express the trauma we experienced or projected. Without directly calling someone out, there is space to collectively work through the harm they may have perpetuated. We now turn to our final scenario which was re-told by Amanda and took place at a local Starbucks.

### 5.5. Scenario 3: Standard Language, Allyship and “Listening to Divergent Viewpoints”: Starbucks

Scenario 3 also came from the February 2022 Forum Theater group. In this scenario, Se-ri (pseudonym) from South Korea shared an experience she had as a customer at Starbucks. The scene began when she was ordering and was asked to repeat herself many times because the barista could not (or would not try to) understand her. This resulted in people behind her in the line getting frustrated, which became the *moment of crisis*. When the scene stopped, spectactors were asked to reflect on what happened and discuss possible solutions. Some of the solutions proposed bystanders speaking up on behalf of Se-ri, while others proposed she respond with humor asking how many languages the barista spoke, or other quips that demonstrated resistance. We now turn to our discussion, which combines examples drawn from our collective discussion with quotes from teacher learners in the group to illustrate further some of the benefits and experiences. We note that some of these data come from previous work reported in Morales et al. (2025).

### 5.6. Implications

In our conversation together about this scenario, Amanda spoke of how when she reenacted the scenario, Se-ri shared that since living in the United States, when she tried to speak in English, many did not quite understand her which ultimately made her shut down, avoid engaging in social situations, and made her feel unmotivated, nervous, and embarrassed to speak. This difficulty with language development increased her awareness of language hierarchies related to native and non-native speakers and made it difficult to make friends (Kiramba et al., 2025/2022). These effects exemplified behavioral and psychological consequences of these kinds of microaggressions.

In written reflections from teacher learners about this scenario, Se-ri’s peers noted that they learned on a personal level why it is important “to be empathetic, understanding, and helpful” to international students/transnational youth because “they took a huge leap coming here to learn something new, and it is our job to help them in every which way we can.” One peer specifically spoke about how they could relate this to their future students and how hard this might be on their students’ self-esteem or confidence to learn and practice their English when there is a threat of these kinds of language-based microaggressions. Another student mentioned how they could connect this to helping them better understand the behaviors of the parents of their international, transnational youth who may feel nervous to speak to their child’s teacher or may even avoid engaging with the school all together. A different student said these experiences deepened their “understanding for how their lives here are impacted by the way we [native English speakers] treat them each and every day”. Another indicated how these more nuanced counter-stories and varied scenarios about language, nativity, accent and religious biases helped her to see how “racism/discrimination for them does not just occur between black and white individuals, but between Asian Americans, Chinese Americans, and other cultural groups as well.” In another written reflection, a student shared that she now realized that transnational youth and people of Color:

*… don’t need a ‘white hero’ to come and save them from someone discriminating against them. They often are more than capable of standing up for themselves, but they need the social support to be able to do so without backlash. As a white person, they don’t need me to speak for them but rather to stand with them.*

This was a point that Tianna had made about how the Forum Theater fomented good “allyship” and Brendan pointed out how the arts-based activities allowed teacher learners to understand the importance of dialog and not avoiding these topics. In stopping to “smell the roses”, teacher learners also had to get their hands dirty and “get messy” Brendan said. Theresa remembered how a teacher learner had asked during the workshop if she should “step in in situations like this” to support her friend. Concerned with being perceived as the ‘White savior’, she was not sure what to do. Hence, this led to a really good conversation in which Amanda (also co-leading the workshop) shared with the group how people of Color like herself experienced these kinds of things so much, they sometimes get tired of having to stand up for themselves and appreciate someone else doing it for a change. Crystal also added in the reflection session that this is why the warm-up and getting-to-know-you activities were so essential to these kinds of workshops being successful, because “You can’t just come into a group and start sharing these… we really have to set the scene”. This led us to discuss the humanizing elements of the workshops and especially the warm-up exercises that help “build the bond” (Crystal) and foster authentic connections early on in the workshop.

Returning to student participants in the Forum Theater, Amanda remembered how in written reflections, some of them talked about arts-based activities more generally, and how they “force you to participate”. They make the “actions come alive, and they make the lesson hold true meaning”. One said “I was more closely able to understand how international students felt and really juggle what could have been done. It opened my mind and committed it to memory.” Another student said that she able to practice for these kinds of situations. Instead of being a bystander, [she] now knows how to “appropriately join in on the conversation and stand up for the person.” She indicated that before engaging in this experience she “didn’t know how [she] could stand up for someone without getting myself into more trouble, but now [she] understands” and “has more empathy”. Another described the experience as not only offering an “opportunity to meet new people who have had completely different experiences than I have” but also said she could

*… see things from their perspective. A few members in my group experience racial microaggressions frequently, so hearing their firsthand experiences, although it is heartbreaking, allowed me to see just how harmful these comments can be.*

A different student mentioned how she “thought that this experience was beneficial because we as people are much closer than we think, but our prejudice and judgments can cloud our thoughts.” She further described the importance of teachers being knowledgeable and willing to act. She said, “When teachers understand and interact with different cultures, they can prevent prejudice and judgments from harming a student’s educational path.” Another student talked about how this experience with Forum Theater challenged some of the assumptions she had about doing this kind of activity within a large and highly-diverse group. She said:

*… many of the situations which were acted out during the activity could (unfortunately) get some people started on controversy, rather than acknowledging them as a violation of human decency. However, as I looked around the room, I realized that every single person was not looking to debate one another, rather listening to divergent viewpoints and reexamining what each individual believes to be true.*

A final participant noted how nervous she was to share her experience at first because in the past, people would just simply ignore or say they experienced the same thing and to ‘get over it’, “but this group was different.” Amanda considered this comment in our reflection session adding:

*… That line hit me hard and just really speaks to the importance of appropriately setting the context for this kind of deep, vulnerable and humanizing work to be most meaningful.*

This scenario demonstrates how processing and co-generating responses to real life contextualized experiences deepened teacher learners’ empathy for transnational students facing language-based microaggressions, highlighting their psychological and social impacts and prompting learners to reconsider allyship and recognize the humanizing conditions needed for honest dialogue, shared vulnerability, and meaningful action.

## 6. Conclusions

### 6.1. Summary of Findings

This study has shown the incredible potential of transdisciplinary arts-based approaches to teaching and research for communicating a holistic understanding of the language, education, and identity development of transnational youth, and for better preparing teachers to value and capitalize on their linguistic and cultural resources. Our first scenario described a scene in which a student was not allowed to help a classmate, and the teacher operated under monolingual ideologies that did not recognize the funds of knowledge of the students nor help the learners feel welcome. Our reflection revealed how the teacher learners (also co-authors of this paper) were able to develop critical consciousness about how their own actions as teachers could be damaging or discriminatory while learning strategies for valuing and harnessing the funds of knowledge of students such as Brendan, who had bilingual skills that could have helped both the teacher and student. In our second scenario, we discussed a microaggression in which a student wearing a hijab was assumed to speak Arabic by a bus driver who could not accept that she could identify as Muslim without being able to speak Arabic. Here we pondered the sense of belonging that transnational youth often struggle with (as noted by Przymus & Serna Gutiérrez, 2022) as they renegotiate their multilingual transnational identities when microaggressions questioning their ‘Americanness’ occur. Our last scenario enacted a scene at Starbucks in which a student had to repeat her order multiple times which resulted in shaming by other customers in the line. In this scenario, teacher learners shared how they gained knowledge of allyship as part of their continuing development of critical consciousness. Through this process, they also developed empathy and deepened their understanding of what transnational youth experience such as when the student was asked to repeat her order over and over.

### 6.2. Implications for Practitioners and Researchers

Our study uncovered immense benefits of arts-based approaches to help teacher learners see student experiences from different perspectives, be comfortable with uncomfortable topics and be more likely to engage in these conversations rather than avoiding them in the future. All in all, the arts-based approach facilitated the cultivation and nurturing of the linguistic, educational, and identity resources of students, while equipping them with practical tools and language to explore solutions for discrimination, stereotyping and racism in school situations. We believe that teacher education programs have much to gain from embodied approaches like those described in this study, which open up spaces for critical dialog and reflection on teaching practices. Moreover, they can elevate transnational youth in ways that position them as experts (Kwon, 2022) that we can all learn from.

In terms of research, we believe that non-traditional arts practice research like ours can dive much deeper into the messy, complex (and beautiful) spaces that transnational youth inhabit, unearthing more holistic and asset-based ways of helping transnational students feel welcomed, comfortable, and more agentic within schooling environments. These approaches are better positioned to answer our research questions related to psychological, cognitive and behavioral decisions and their impacts. Rather than looking at what is ‘lacking’ in these students, this type of research makes us ask, what is lacking in us? How can we as a society make schools a place where globally mobile students can thrive? How can we change our own behavior and thinking to make school somewhere these students feel at home, wherever they are? We also believe our work shows how creative projects like Forum Theater are open-ended. That is, we never know what the participants will come up with in their scenarios, but they are always based on their own embodied experiences. Dialoging about those experiences and reflecting on them together through collective autoethnography was a valuable way to make sense out of them, and learn from them, and we hope that teacher educators reading this will share our findings with their own students so that it becomes even more valuable.

### 6.3. Concluding Thoughts

We conclude with a nod to the work of Gándara and Jensen (2021), who refer to transnational students as ‘los alumnos que compartimos’ [the students we share]. As Hamann et al. (2013) point out, these words assert the common ground that transnational youth are a shared concern for *all* teachers the moment they come to school. Therefore, we need to continue the important work of preparing teachers in this area, and furthering research about the power of arts-based approaches as a mechanism to provide more holistic teacher education opportunities regarding transnational youth.

## Figures and Tables

**Table 1:** Scenario Ledger.

Scenario #	Descriptive Title	Workshop Iteration	Data Sources Analyzed
1	Classroom/teacher and students	7 June 2025	Reflection session transcript
2	Bus/student with hijab	22 February 2022	Reflection session transcript
3	Student ordering at Starbucks	22 February 2022	Reflection session transcript
			written reflections

## Data Availability

The data presented in this study are available on request from the corresponding author due to issues of privacy and ethical reasons.
